# Micro- and Nanoplastics in Dentistry: Challenges in Obtaining High-Quality Evidence

**DOI:** 10.3390/ma18184269

**Published:** 2025-09-12

**Authors:** Luka Šimunović, Ivana Bačić, Senka Meštrović

**Affiliations:** 1 Department of Orthodontics, School of Dental Medicine, University of Zagreb, 10000 Zagreb, Croatia; 2 Forensic Science Centre “Ivan Vučetić”, Ministry of the Interior, 10000 Zagreb, Croatia; ivana.bacic@mup.hr

**Keywords:** microplastics, nanoplastics, dentistry, dental materials, environmental exposure, occupational health, sustainability, analytical methods

## Abstract

The increasing concern over micro- and nanoplastic (MNP) pollution has extended into the field of dentistry, where polymer-based materials and clinical procedures may contribute to environmental and occupational exposure. This narrative review aims to synthesize current knowledge on MNPs in dentistry and identify gaps that hinder high-quality evidence generation. Methods include a critical appraisal of existing literature across dental disciplines, including orthodontics, restorative dentistry, and prosthodontics, with emphasis on experimental designs, sampling strategies, and analytical methods. Results reveal that while in vitro studies suggest measurable particle release from common dental materials, real-world exposure data remain sparse, especially regarding airborne and ingested microplastics. Furthermore, inconsistencies in study design, lack of standardized detection methods, and underrepresentation of clinical settings limit the generalizability of findings. This review highlights that while micro- and nanoplastic release from dental materials is evident in laboratory studies, real-world exposure data remain limited and inconsistent. To advance the field, harmonized research protocols, interdisciplinary collaboration, and standardized detection methods are urgently required. Practical measures, such as improved clinical practices and sustainable material choices, can already help reduce emissions. By outlining both current knowledge gaps and actionable strategies, this work provides a foundation for informed decision-making in clinical, regulatory, and environmental contexts.

## 1. Introduction

Plastics are ubiquitous in modern dentistry, from disposable clinical supplies to durable restorative materials. With the global proliferation of plastic waste, there is rising concern that dentistry contributes to microplastic (<5 mm) and nanoplastic (<1 µm) pollution that may harm patients and the environment [1,2]. Every piece of plastic ever produced still exists in some form, breaking down over time into smaller fragments that persist in ecosystems [3,4]. Dentistry’s reliance on resin-based restoratives, polymeric prosthetics, and single-use plastics means that a portion of this plastic eventually degrades into microscopic particles [5]. Recent estimates warn that oceans could contain more microplastics than fish by 2050 [6], and healthcare activities—including dentistry—are under scrutiny for their plastic footprint [1].

Microplastics and nanoplastics (MPs/NPs) are not only an environmental issue but also a human health concern. These particles can adsorb and carry toxic chemicals and microbes, and their minute size allows them to be ingested or even inhaled. In the oral cavity, mechanical and chemical forces can induce the release of MPs/NPs from dental materials [2]. Unlike obvious clinical hazards, the effects of chronic exposure to these invisibly small particles are subtle and hard to trace. High-quality evidence on their prevalence and impact is limited, as detecting and quantifying MPs/NPs in biological or environmental samples is technically challenging [7,8]. Nevertheless, emerging data from toxicological studies indicate that MPs/NPs can elicit inflammation, oxidative stress, and immune dysregulation [7,8]. While direct dental evidence is limited, studies on resin degradation products suggest similar pathways may be relevant in the oral environment [9,10].

Previous reviews have outlined the environmental burden of dental plastics [1]. This review builds on that foundation by providing a critical, narrative synthesis that not only collates current evidence but also examines why robust data are scarce, identifies methodological barriers to accurate detection, and proposes strategic priorities for future research. By doing so, we aim to clarify the state of knowledge on MPs/NPs in dentistry and chart a path for clinically and environmentally relevant investigation.

## 2. Sources of MP/NP Particles in Dentistry

Plastic particles in dentistry originate from a wide range of sources, broadly categorized into primary MPs (manufactured micro-sized ingredients) and secondary MPs (particles generated by degradation or wear of larger plastic items) [5]. Dentistry contributes to both types:

### 2.1. Restorative and Prosthetic Materials

Resin-based composites, sealants, and denture polymers can shed microscopic debris during placement, finishing, or routine wear. In clinical simulations, grinding or polishing a composite restoration produces abundant microparticles with median sizes on the order of 5–10 µm [4]. These particles have extremely high surface area (~103 m^2^/kg), which can promote the leaching of unreacted monomers and additives [4]. A recent study confirmed that everyday dental procedures like removing old composites (“secondary MPs”) generate significant plastic dust (20–100 µm diameter) that settles on surfaces throughout the operatory [8]. Even passive degradation plays a role: polymer-based prostheses (e.g., acrylic dentures) release small particles during simulated mastication and cleaning, albeit in modest quantities per day [8]. Over months of use, however, these releases may accumulate in the oral cavity or gastrointestinal tract. Consequently, patients with long-standing dentures or extensive composite restorations may experience chronic exposure, although the clinical significance of such exposure remains unclear due to a lack of longitudinal in vivo data.

Non-polymeric prosthetic and restorative materials also warrant consideration. Although zirconia, ceramics, and amalgam are not polymers and thus do not release MPs/NPs in the same sense as resin-based materials, their processing and clinical handling can nonetheless generate particulate debris. For instance, CAD/CAM (computer-aided design/computer-aided manufacturing) milling of zirconia and lithium disilicate ceramics produces airborne and waterborne particles in the micro- and nanoscale range, which have been detected in laboratory environments and slurry waste streams [11]. Similarly, chairside grinding and polishing of zirconia and porcelain restorations releases respirable ceramic dust that may be inhaled by dental staff if not adequately controlled [12].

Amalgam presents a distinct environmental concern due to mercury release, but it can also generate metallic particulate waste during removal or polishing [13]. While such debris is not classified as MPs/NPs, it contributes to the broader issue of dental particulate pollution. Taken together, these examples indicate that prosthodontic materials of all classes—polymeric and non-polymeric—can contribute to particle release at different stages of their life cycle.

### 2.2. Orthodontic Appliances

Thermoplastic clear aligners have drawn attention as a source of MPs. In vitro aging of aligners in artificial saliva showed that routine mechanical stress (simulating 1–2 weeks of wear) causes detachment of polymer fragments approximately 5–20 µm in size [14,15,16]. Raman spectroscopic analysis identified the aligner polymers (polyethylene terephthalate in many brands, polyurethane in others) in the released particles [14], confirming they are derived from the aligner material itself. The highest particle release was observed in certain aligner brands, suggesting material formulation and manufacturing influence particle generation [14]. Notably, orthodontic adhesive resins can also produce MPs: bracket debonding has been shown to create particulate debris capable of estrogenic activity in vitro [17], likely due to Bisphenol-A-derived monomers in the adhesive. These findings raise concerns that routine orthodontic procedures might expose patients (especially adolescents) to endocrine-disrupting plastic byproducts [18].

### 2.3. Preventive and Oral Hygiene Products

Some toothpastes and polishing pastes historically contained microbeads (MPs typically made of polyethylene) as abrasives. Although such ingredients are being phased out in many jurisdictions, legacy products and certain cosmetics still contribute to primary MPs. Polyethylene microbeads (~<1 mm) have been added to toothpastes to enhance polishing and whitening [19]. Concentrations up to ~1–2% by weight have been reported in commercial pastes [20]. These MPs inevitably get flushed into wastewater during toothbrushing, bypassing most water treatment and ending up in aquatic environments. In one estimate, India’s annual release of MPs from toothpastes was 1.4 billion grams [21], highlighting the magnitude of seemingly minor sources when aggregated across large populations. Other daily use products like mouthwashes sometimes contain polymer thickeners or capsule beads, and while their contribution to MPs is less studied, they add to the cumulative load. Toothbrushes and dental floss, made of nylon, polyethylene, or PTFE (polytetrafluoroethylene) fibers, are another concern. Bristle wear during brushing can shed microscopic nylon fragments directly into the oral cavity or drain [1]. Used floss filaments might fragment, especially those with multifilament strands, releasing tiny fibers. Though quantitative data are sparse, the sheer volume of plastic oral care items disposed of annually (billions of toothbrushes and floss picks) suggests a non-trivial generation of secondary MP.

### 2.4. Clinical Supplies and Equipment

Single-use plastics in dentistry (gloves, suction tips, barriers, syringe tips, etc.) contribute indirectly to MP pollution upon disposal and degradation. The COVID-19 pandemic intensified use of disposable personal protective equipment (PPE) in dental clinics [22]. Masks, gowns, and gloves are often made of polymers (polypropylene nonwovens, nitrile, etc.) that fragment over time. While PPE-related MP release mostly occurs in landfills or the general environment [23], there is potential for indoor fragmentation—for example, friction on a face mask shedding microfibers that could be inhaled by dental staff. In fact, a 2024 study of dental clinics found significant MP deposition on indoor surfaces, and the authors suggested improved ventilation and limiting plastic PPE where safe to do so [24]. Even durable equipment can be a source: high-speed handpiece tubes, compressor lines, and vacuum hoses made of plastics might abrade internally, though this is an area requiring more research.

### 2.5. Waste Management Pathways

The fate of dental plastics after use can generate MPs at a remove from the clinic. For example, improper disposal or recycling of dental plastics (such as clear aligners or plastic packaging) can lead to environmental degradation into MPs. Dental laboratories grinding acrylic models or CAD/CAM milling blocks produce plastic shavings that, if not properly captured, can enter wastewater. A systematic survey in Pakistan’s dental hospitals found MP pollution in clinic dust and wastewater, with PET (polyethylene terephthalate) and other plastics present from sources like dental uniforms, patient bibs, and acrylic scraps [24]. Thus, the “downstream” plastic waste from dentistry—much of it not traditionally considered biomedical waste—is an emerging contributor to MP contamination in the environment [25].

## 3. Analytical Methods for Detecting Dental MPs/NPs

Sensitive and specific analytical methods are required to identify MPs/NPs amidst complex dental matrices (saliva, dental materials, clinic dust). However, traditional analytical tools each have limitations when applied to microscopic plastics [26]. Table 1 provides a comparative overview of major methods used to detect and characterize MPs/NPs relevant to dentistry, highlighting key parameters. These include spectroscopic methods (FTIR (fourier-transform infrared spectroscopy) and Raman microscopy), microscopic imaging (electron microscopy), and thermal analytical chemistry (pyrolysis-GC-MS). Researchers often employ a combination of techniques to compensate for individual limitations, for example, using microscopy to count particles and spectroscopy to identify polymer types [27,28,29].

### 3.1. Fourier-Transform Infrared Spectroscopy (FTIR)

FTIR microspectroscopy is a workhorse for MP identification. It detects the vibrations of chemical bonds, yielding characteristic spectra for different polymers. Attenuated total reflectance micro-FTIR can analyze particles down to ~20 µm, or even ~10 µm with specialized setups [27,30,31,32]. Every common dental polymer (e.g., polymethyl methacrylate in dentures, Bis-GMA (bisphenol A-glycidyl methacrylate)/TEGDMA (triethylene glycol dimethacrylate) resins in composites, polyethylene in microbeads) has a unique IR fingerprint, allowing high polymer specificity. FTIR works best on isolated particles deposited on an IR-transparent substrate or filter. In practice, a sample (saliva, water, dust) must be filtered or dried, since water and other constituents interfere with IR absorption. The method is non-destructive and can even be used on particles embedded in a thin section (for instance, MPs within tissue biopsies), though strong IR absorption by the matrix can complicate analysis. Modern FTIR imaging systems with focal plane array detectors can scan entire filters, mapping thousands of particles automatically—a boon for throughput, with some systems analyzing a sample in under an hour. The cost of an FTIR microscope system is relatively high, but it is available in many laboratories, and databases of polymer spectra are well-developed. A noted limitation is the detection size floor: standard FTIR cannot reliably detect nanoplastics, because the wavelength of mid-IR light (~10 µm) exceeds the particle size [7]. Thus, FTIR effectively covers the MP range (tens of µm and up) but misses nanoparticles. Additionally, densely overlapping particles or composite particles (containing mixture of polymers or fillers) can yield convoluted spectra that are hard to interpret.

### 3.2. Raman Microspectroscopy

Raman spectroscopy complements FTIR by detecting molecular vibrations via inelastic light scattering. Using visible or near-infrared lasers focused through a microscope, Raman can analyze much smaller particles—on the order of 1 µm, limited mainly by the optical diffraction limit [33]. Indeed, Raman’s smaller laser spot size grants better spatial resolution, allowing detection of MPs “as tiny as a few micrometres” and in some cases even sub-micron if using specialized techniques (surface-enhanced Raman, etc.) [34]. Raman spectra likewise provide high polymer specificity, and in certain cases, Raman can distinguish polymer additives or crystallinity. A practical advantage is that Raman can analyze particles on virtually any substrate (even glass slides or metallic filters, which are opaque in FTIR). It is also less affected by water content, so damp samples or hydrated hydrogels can sometimes be analyzed directly [35]. However, Raman spectroscopy has its own challenges: fluorescence from dyes or organic matter in samples can overwhelm the Raman signal, a relevant issue since dental composites often contain pigments and fluorescent agents. High laser power can also burn or alter plastic particles, especially dark-colored ones, confounding analysis [36]. In terms of throughput, conventional point-by-point Raman mapping is slow—scanning a filter for hours to locate tiny particles. Emerging high-throughput Raman imaging systems (using wide-field or automated mapping) are improving speed [37], but generally, Raman is considered lower-throughput than FTIR for MPs. The cost of a research-grade Raman microscope is comparable to or higher than that of FTIR, and it similarly requires an experienced operator. Despite these hurdles, Raman is invaluable for detecting the smaller MPs that FTIR misses, and many studies combine both techniques for comprehensive analysis [38,39,40].

### 3.3. Scanning Electron Microscopy (SEM)

SEM is widely used to visualize the morphology of MP/NP particles. It can magnify objects to the nanoscale, readily imaging particles well below 1 µm that are beyond optical resolution. For dental materials, SEM has shown the roughness of composite and polymer wear debris or the fibrous nature of mask microfibers [41]. Detection range is essentially limited by instrument resolution (tens of nm) and the ability to discern particles against a background. In one study, SEM revealed aligner-derived particles ~5 µm in size and also confirmed their presence on filters alongside spectroscopic analysis [14]. However, SEM by itself does not identify polymer composition—it only provides size, shape, and topographical details [42]. Many SEM systems are coupled with energy-dispersive X-ray spectroscopy (EDS), but EDS detects elemental content (e.g., presence of Si from fillers or Cl in PVC (polyvinyl chloride)) rather than polymer type. As a result, SEM is often used in conjunction with FTIR/Raman: first, SEM images the particles and perhaps counts them, then spectroscopic analysis is performed on the same sample spots to identify the polymer. SEM’s sample requirements include thoroughly dry specimens (as it operates in vacuum) and typically a conductive coating (a thin metal film) on insulating plastic particles to prevent charging. This means biological matrices (saliva, tissue) must be removed or sufficiently fixed and dried—raising contamination considerations. Throughput is low to moderate; while automated particle-sizing software exists, analyzing large numbers of particles by SEM is time-intensive, and only surface-exposed particles on a stub or filter will be seen. The instrument cost and maintenance (vacuum pumps, electron source) are high, and operation usually requires specialist training. Despite these constraints, SEM remains a powerful tool for nanoplastics in particular—one of the few ways to actually confirm the presence of nanoparticles visually. Researchers have used SEM to detect ~100 nm particles from degraded plastics in controlled settings [7], though distinguishing them from artifacts can be difficult.

### 3.4. Pyrolysis–Gas Chromatography–Mass Spectrometry (Py-GC-MS)

Py-GC-MS takes a completely different approach by destroying the sample to reveal its chemical signature. In this technique, a sample (containing plastic particles, among other things) is heated to high temperatures (500–700 °C) in an oxygen-free environment, causing polymers to break into characteristic smaller molecules (pyrolyzates). These fragments are separated by GC and identified by MS, producing a fingerprint that can be matched to known polymers [43]. Py-GC-MS is highly polymer-specific and very sensitive—even nanogram quantities of a particular polymer can be detected if present. It excels at identifying and quantifying polymer types in complex mixtures, and is often used to measure total plastic content in environmental samples (e.g., how many µg of polyethylene per gram of sediment). For dental applications, Py-GC-MS could, for instance, determine if MPs in clinic dust are predominantly PMMA (polymethyl methacrylate) (from dentures) or polyethylene (from wipes or barriers). Its great advantage is that particle size is irrelevant—even nanoscale plastics are detected as long as their mass is there. This makes it critical for studying nanoplastics, which elude microscopy [44,45,46]. However, Py-GC-MS does not provide particle count or size information, only mass of polymer types. To address this, it is often complemented by FTIR microscopy, which enables rapid screening of particles down to ~20 µm, and by Raman microspectroscopy, which can identify smaller particles (~1 µm) with high specificity [37,47]. FTIR offers speed and established spectral libraries but limited resolution, whereas Raman achieves higher spatial resolution but is slower and sensitive to fluorescence interference [48,49]. Another limitation is that if a sample contains mixed polymers, their pyrolysis products overlap, complicating interpretation (though advanced data analysis can deconvolute multiple polymers).

Py-GC-MS can handle matrices like dental dust or sludge after minimal preparation (drying or digesting away non-plastic organic matter). In practice, one often performs a chemical digestion (to remove biological material) and filtration to concentrate plastics before pyrolysis [7]. The method is destructive (samples cannot be re-analyzed) and requires careful calibration to relate signal to polymer mass.

Cost and throughput: A dedicated pyrolyzer unit attached to a GC-MS is expensive and typically found in specialized analytical chemistry labs. Each run might take 30–60 min, so throughput is on the order of perhaps dozens of samples per day. For answering broad questions like “how much plastic does a procedure generate?” Py-GC-MS is extremely useful; for pinpointing individual particles, it must be paired with microscopic methods.

### 3.5. Other Methods

A variety of additional techniques are emerging for MP/NP detection, though not yet routine in dental research. For nanoplastics in liquids, dynamic light scattering (DLS) and nanoparticle tracking analysis (NTA) can estimate particle size distributions down to the hundreds of nanometers, but they cannot tell polymer composition and are easily confounded by heterogeneity [26]. Fluorescence staining (e.g., Nile Red dye) can label hydrophobic plastic particles to aid visual counting under a microscope, an approach used in environmental monitoring—its applicability to dental samples (which often contain other hydrophobic debris) is limited but worth exploring. Advanced hybrid methods like AFM-IR (atomic force microscopy coupled with infrared spectroscopy) can directly measure chemical spectra of nanoscale particles [50,51], and laser-based techniques like MALDI-ToF (matrix-assisted laser desorption/ionization time-of-flight) mass spectrometry are being tested for nanoplastic identification [42]. However, these advanced methods require further development to handle real-world samples and are not yet common in the dental materials literature.

**Table 1:** Comparison of analytical methods commonly used to detect and characterize MPs/NPs relevant to dentistry.

Method	Detection Size Range	Polymer Specificity	Sample Matrix Compatibility	Relative Cost (Instrumentation)	Throughput (Samples or Particles)
FTIR Microspectroscopy (μFTIR)	~10 µm and above (MPs); not suitable for <10 µm NPs	High—identifies polymers via unique IR spectra. Limited for mixtures or very small particles.	Works on dry, isolated particles (filters, surfaces). Water or thick matrices interfere; requires sample prep (e.g., filter water or digest tissue).	High (FTIR microscope ~$100 k USD). Widely available in labs; moderate operating cost.	Moderate–High: Automated mapping can analyze thousands of particles per sample in hours. 1 sample/hour is typical for imaging a filter.
Raman Microspectroscopy	~1 µm and above (can detect smaller MPs than FTIR). Some techniques push to sub-µm; NPs remain challenging.	High—distinct Raman spectral “fingerprints” for polymers. Fluorescence or additives can interfere.	Works on dry or wet samples; can analyze particles on many substrates (including glass). Minimal matrix interference except fluorescence.	High (Raman microscope ~$100–200 k). Specialized instrument, high expertise needed.	Low–Moderate: Point mapping is slow (many minutes per particle or field). Modern imaging Raman can speed up, but is generally slower than FTIR.
Scanning Electron Microscopy (SEM)	~100 nm and above (visual detection). Excellent for morphology of micro- and nanoplastics.	None by itself—images only. (With EDS: elemental info, but not polymer type).	Requires dry, solid samples; conductive coating often needed. Incompatible with high moisture or thick organic layers (must remove/digest matrix).	High (SEM ~$200 k, plus maintenance). Typically available in central facilities; operator required.	Low: Each sample requires manual prep and scanning. Dozens of particles can be imaged in a session, but large-scale quantification is very time-consuming.
Pyrolysis GC–MS (Py-GC-MS)	No lower size limit (detects polymers in any size down to nanoplastics, given sufficient mass).	High—polymer types identified by characteristic pyrolysis products. Can also detect polymer additives.	Tolerates complex matrices after minimal prep (drying/cleanup). Solid, liquid, or even tissue digest can be analyzed; non-plastic organic matter should be removed to avoid interference.	High (GC-MS ~$150 k + pyrolyzer unit). Needs skilled analytical chemist; consumable costs per run.	Moderate: ~30–60 min per analysis. Can process ~10–20 samples/day on one instrument. Yields bulk data (polymer mass), not particle counts.

“Detection Size Range” indicates approximate lower size limits for particle detection. Polymer specificity denotes the ability to distinguish polymer types. Matrix compatibility notes what sample types or preparations are required. Cost and throughput are relative estimates to provide a sense of practical feasibility. References: [7,43,46,50,51].

In applying these methods to dentistry, researchers must often combine approaches. For example, to study particles released during composite grinding, one might collect the debris on a filter, use SEM to examine particle morphology and size distribution, then perform μFTIR or Raman on selected particles to identify their polymer composition [14]. For assessing human exposure (patient ingestion or staff inhalation), Py-GC-MS might quantify total polymer uptake, while microscopy pinpoints the particle sizes likely to deposit in specific organs. Each method’s biases also need to be managed. Spectroscopy might miss black or highly irregular particles; SEM might over-count harmless dust fibers as “microplastics” unless validated by spectroscopy. Analytical quality control—including clean blanks to avoid sample contamination by ubiquitous environmental MPs—is essential to obtaining trustworthy data. Indeed, one reason evidence is hard to gather is that measuring MPs at trace levels is an analytic feat in itself. Advances in detection (e.g., improved nanoplastic sensors, higher-throughput imaging) are continually improving our ability to study these particles, but currently available methods still have significant blind spots.

## 4. Contamination and Confounding—Critical Controls

MP/NPs are so widespread in the modern environment that they pose a serious challenge to research integrity, particularly in dentistry. Without strict contamination controls and well-defined strategies for attributing exposure, both laboratory and observational studies risk producing misleading or irreproducible findings.

### 4.1. Laboratory Contamination: Avoiding False Positives

Microplastic contamination in laboratory settings is difficult to avoid without well-enforced protocols. Airborne fibers from clothing, ventilation, and even paper towels can settle on samples, filters, or analytical surfaces—posing a particular risk in FTIR and Raman spectroscopy, where stray fibers may be misidentified as sample particles.

To reduce contamination, work should be conducted under laminar flow hoods or clean air benches (International Organization for Standardization (ISO)-classified). Non-synthetic lab coats and gloves help reduce fiber shedding, while glass or metal containers are preferred over plastic, which may release particles. Solvents and rinse water should be pre-filtered (e.g., using 0.2 µm PTFE or glass fiber filters), and blank controls—including procedural blanks and airborne deposition dishes—should be run with every experiment. Plastic consumables, when used, must be validated to ensure they do not contribute to sample contamination [52,53].

Contamination controls should always be reported in publications to ensure transparency and reproducibility.

### 4.2. Confounding in Exposure Attribution

In human studies, MP/NP exposure comes from multiple sources simultaneously—air, food packaging, drinking water, textiles, household dust, and cosmetics all contribute to the overall burden [54]. This makes it difficult to isolate the contribution from dental materials unless background exposure is carefully accounted for.

For instance, finding PET in fecal samples of a patient wearing aligners could stem from the aligners themselves, but also from diet or ambient exposure. To address this, researchers should prioritize polymers that are unique to dental materials (e.g., Bis-GMA, TEGDMA, PMMA), as these are less likely to appear from environmental sources. Stable isotope labeling, such as deuterated monomers in resins, offers a way to trace sources more reliably [55].

Collecting detailed exposure histories (including diet, job, and home environment), along with using matched controls and repeated sampling, helps reduce variability and improve the specificity of results, and matched control cohorts and repeated measures are essential to account for day-to-day variability in MP excretion or inhalation.

Ultimately, high-quality MP/NP research in dentistry demands rigorous contamination prevention and thoughtful source attribution. Without these, even technically advanced studies may yield uncertain or unconvincing conclusions. The field should move toward standards similar to those used in trace contaminant toxicology and clinical pharmacokinetics.

## 5. Exposure Pathways and Biological Effects

### 5.1. Exposure in Dentistry

The primary routes of exposure to dental MPs are ingestion and inhalation. Patients can ingest MPs shed from dental materials in their mouth, while dental professionals might inhale microplastic-laden dust during procedures. Ingestion by patients may occur with orthodontic aligners, dentures, and resin-based restorations. For instance, a patient wearing clear aligners continuously will swallow saliva that contains MP fragments abraded from the aligner [14]. Similarly, micro-debris from a freshly polished composite filling can mix with saliva and be swallowed. Over long periods, even low-grade release from dentures (PMMA particles) or sealants might contribute to oral ingestion. Once swallowed, some particles pass through the gastrointestinal tract and are excreted, but studies in other fields show a fraction can be absorbed, resulting in varying underlying problems like tissue inflammation, abnormality in the gut microbiome, and disrupted endocrine and DNA damage [2,56,57,58]. MPs around 20 µm or larger likely pass transiently, whereas nano-sized plastics (<1 µm) have the potential to cross cell membranes and enter the bloodstream [1]. Inhalation by staff (and patients) is a concern during high-speed dental drilling, sanding of acrylics, or any aerosol-generating procedure involving polymers. The dust generated by grinding composites or dentures includes MP particles in the respirable size range (<10 µm) [8]. A recent exposure study in dental clinics measured an average daily inhalation of ~20–30 MP particles per person in a busy dental operatory [24]. These were mostly polyethylene terephthalate (PET) particles and other common polymers, found settling on surfaces and presumably in the air [24]. Interestingly, that study noted female staff had slightly higher estimated inhalation (possibly due to different working positions or use of makeup that attracted fibers) [24]. Inhaled MPs can deposit in the airways; larger particles may be cleared by cilia and swallowed (contributing again to gut exposure), while smaller ones might lodge in the lungs.

**Figure 1:**
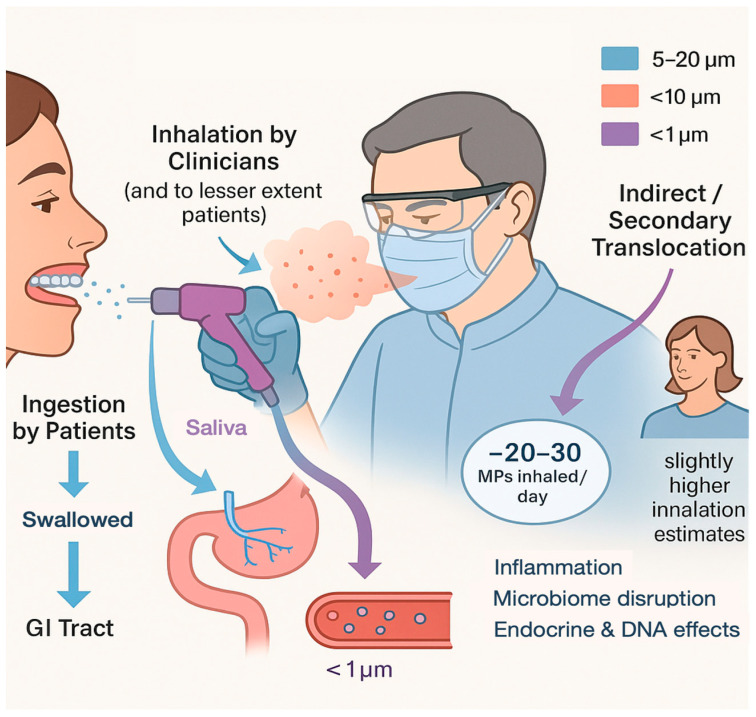
Microplastic exposure in a dental setting involves three intersecting pathways: ingestion by the patient, inhalation by staff and patients, and potential nanoparticle translocation. Patients may ingest MPs released from aligners, dentures, or polishing debris (~5–20 µm). Dental clinicians and nearby patients can inhale respirable MP dust generated during polymer-based procedures (<10 µm), estimated at ~20–30 particles/day in busy operatories, predominantly PET and related polymers. Smaller nanoplastics (<1 µm) may cross mucosal or cellular barriers, although direct dental-specific evidence remains lacking. Some inhaled MPs may deposit in airways and be cleared by mucociliary action—eventually swallowed, leading to secondary ingestion.

Another subtle pathway is translocation through mucosa. Nanoplastics might possibly penetrate oral mucous membranes or open dentinal tubules during dental treatments, although direct evidence is lacking. Given that nanoparticles are used intentionally in some dental materials (e.g., nanofillers in composites), one cannot rule out that nanoplastic byproducts might interact at the tissue level if released. Overall, actual exposure levels in dentistry remain hard to quantify with precision, but the presence of MPs/NPs in dental environments is now documented [8,23]. Even if dental sources are a relatively small contributor compared to food or water MPs, they represent a direct exposure route in a vulnerable area (oral cavity) and thus merit attention (Figure 1).

### 5.2. Toxicological and Health Implications

The health risks of micro/nanoplastics related to dentistry are extrapolated largely from toxicology studies in medicine and environmental health, as direct clinical data are scarce. Nonetheless, several plausible concerns have been raised.

#### 5.2.1. Local Tissue Reactions

The oral tissues (gingiva, mucosa) and respiratory tract can mount inflammatory responses to foreign particles. In vitro cell studies provide some evidence: one study found that MP particles generated during dental procedures induced inflammatory cytokine release in macrophages and caused oxidative stress in oral epithelial cells at realistic exposure doses [8]. Such responses, if they occur in vivo, could hypothetically contribute to mucosal irritation or local immune modulation. For example, chronic gum contact with MP debris might exacerbate irritation or act as an adjuvant for other irritants. However, these effects have not been demonstrated in patients yet—no clear cases of “microplastic gingivitis” or similar. The lack of overt clinical signs is not surprising given the low levels, but subtle chronic inflammation is possible.

#### 5.2.2. Systemic Uptake and Effects

Once ingested or inhaled, MPs may have far-ranging effects. Particles under ~20 µm can penetrate deeper into tissues; indeed, gut absorption of the smallest fraction of MPs has been reported in animal studies [59,60]. The Minderoo-Monaco Commission on Plastics and Health [61] concluded that plastic pollution is linked to immune system stress, oxidative damage, and even cancer risk in the broader population. MPs in the gut have been associated with dysbiosis and inflammation in rodent models, raising questions about impacts on human gastrointestinal health [62]. Over prolonged use of dental devices, gradual ingestion of MPs could contribute to cumulative exposure, although the actual quantities and health significance remain uncertain. Laboratory studies suggest that some particles may translocate to internal organs such as the liver, kidney, or even cross the placenta [63,64]. While the possibility of long-term effects, including inflammation-related conditions, is under discussion, no direct evidence currently links dental-origin MPs to systemic disease or cancer. This underscores the need for cautious interpretation and further research.

#### 5.2.3. Chemical Leachates (Indirect Effects)

Plastics often carry additives—bisphenol A (BPA), phthalate plasticizers, flame retardants, pigments—which can leach out. In the confined space of the mouth, leachates from even a small mass of MPs could reach biologically active levels. BPA, in particular, is relevant as it is a component of many dental resins [65]. Saliva studies show that resin-based fillings and sealants release trace BPA during the first 1–2 days after placement [66]. MPs from these materials could continue to release BPA or related bisphenols as they degrade [67]. BPA is a well-known endocrine disruptor associated with reproductive, developmental, and metabolic effects [65,66]. Several studies have confirmed that dental composites and sealants can release trace amounts of BPA into saliva, particularly within the first 24–48 h after placement [66,67]. Moreover, orthodontic adhesive particulates generated during bracket removal have demonstrated estrogenic activity due to residual Bis-GMA- and BPA-derivative monomers [68]. Beyond BPA, other resin components such as triethylene glycol dimethacrylate (TEGDMA) and urethane dimethacrylate (UDMA) have been shown to leach from dental materials under simulated oral conditions, exhibiting cytotoxic or genotoxic effects in vitro [18]. Although leached concentrations are generally low, their persistence and cumulative exposure during long-term orthodontic or restorative treatment raise concerns, particularly in children and adolescents. Therefore, chemical leachates from microplastic and nanoplastic debris represent not only a potential local irritant but also a possible systemic risk, underscoring the importance of further in vivo and longitudinal research.

#### 5.2.4. Microbial Interactions

An often overlooked aspect is that MPs can act as vectors for microbes (the so-called “plastisphere”). In dental unit water lines, for example, MPs fragments might harbor biofilms of opportunistic pathogens. There is evidence from environmental science that bacteria can colonize MP surfaces and even exhibit increased antibiotic resistance [69]. While speculative in the dental context, one could imagine that MPs stuck in gingival crevices might influence plaque composition or that inhaled MPs could carry bacteria into the lungs. Research has shown in vitro that MPs can modulate oral bacterial biofilm formation [58], although concrete clinical implications remain to be explored.

#### 5.2.5. Occupational Exposure Concerns

For dental professionals, repeated inhalation exposure is a potential occupational hazard. Chronic inhalation of any fine particles (dust) can lead to respiratory issues (e.g., pneumoconiosis-like conditions). Plastic particles specifically might cause foreign-body reactions in airways. A quantitative risk assessment from Akhtar et al. [24] evaluated indoor air in dental clinics and concluded the polymer particles posed “minor to medium” risk levels according to a polymer hazard index. The authors still urged preventive measures, like strong ventilation and using wet grinding techniques to minimize aerosolization [24]. Long-term studies on dental personnel do not yet show any pneumoconioses attributable to MPs, but it may be that effects are subclinical (e.g., mild reduction in lung function over decades). The precautionary principle suggests it is wise to control dust exposure now rather than wait for definitive harm proof.

In summary, while definitive clinical harm from dental MPs has not been proven, the biological plausibility of adverse effects is supported by emerging evidence. MPs can cause inflammation and oxidative stress in laboratory settings at exposures comparable to those in a dental clinic [70,71]. They can carry endocrine-disrupting chemicals linked to systemic effects [18]. And they may serve as vectors for microbes or other toxins. Therefore, the issue is on the radar of researchers and regulators. It is important to emphasize that current knowledge is based largely on experimental and cross-disciplinary extrapolations. The field lacks longitudinal clinical studies—for instance, tracking if patients with 20-year-old dentures have higher markers of inflammation or if dental staff have any health differences correlating with MP exposure. Addressing these gaps requires overcoming the evidentiary challenges outlined in the next section.

## 6. Why High-Quality Evidence Is Difficult to Obtain

Despite growing concern, robust evidence on dental micro/nanoplastics lags behind. There are several fundamental challenges that make it difficult for researchers to gather high-quality data and for policymakers to act on it:

### 6.1. Analytical Limitations

As discussed, detecting MPs/NPs at the scales relevant to dentistry (down to sub-micron) is non-trivial. No single method can seamlessly track nanoplastics from source to biological outcome. The need to combine multiple sophisticated techniques means studies become resource-intensive and require interdisciplinary expertise. Many dental schools or clinical research centers lack access to the necessary instrumentation (e.g., micro-Raman or Py-GC-MS), leading to reliance on collaborations. Even with good methods, issues like contamination loom large. Ambient MPs are literally everywhere—in laboratory air, in commercial reagents, on clothing. Distinguishing real signals from background noise demands rigorous controls, which are not always reported or standardized across studies. This partly explains why measurements of MP release can vary widely between studies. For example, one lab might report “minimal” particles from a denture, while another finds an order of magnitude more—differences could arise from analytical sensitivity or contamination rather than the denture itself. The lack of standardized protocols and reference materials for MP analysis exacerbates this inconsistency [72,73,74]. Unlike, say, testing fluoride release (where protocols are well-defined and traceable standards exist), there is no consensus method for measuring “microplastic release” from a dental material. Efforts are underway globally to standardize MP methodologies, but until they mature, the evidence will continue to include studies of uneven quality and comparability.

### 6.2. Complex Exposure Dynamics

Real-world dental exposures are intermittent, chronic, and multifactorial—hard to replicate in a controlled study. Patients are exposed to a trickle of MPs over years, intermingled with myriad other environmental exposures (dietary plastics, dust, etc.). Designing an epidemiological study to isolate the effect of dental MPS (microplastics) would be extraordinarily challenging. Consider: virtually everyone has some exposure to plastics, so finding a truly “unexposed” control group is impossible. Differences in exposure are quantitative, not absolute. We cannot, ethically or feasibly, randomize patients to “receive microplastics” or not. Therefore, we rely on indirect evidence (e.g., does the number of composite fillings correlate with some biomarker?), which can be confounded by many factors. High-quality evidence would ideally come from long-term cohort studies or case–control studies, but none have been conducted specifically for dental MPs. The subtlety of potential effects (e.g., a slight increase in inflammatory markers) would require large sample sizes to detect. Additionally, separating the influence of MPs from their co-released chemicals is difficult—if an effect is seen, is it due to the physical particle or the monomer leaching out of it? Most current studies skirt this by focusing on hazard identification (does X particle cause Y effect in cells?) rather than true risk assessment under clinical conditions. This narrow emphasis risks building an evidence base that highlights theoretical hazards but offers limited guidance on their real-world significance. To advance the field, research must move toward study designs that replicate realistic clinical exposures, capture cumulative low-dose effects, and integrate both biological and environmental endpoints. Translating in vitro findings into clinically relevant risk models is essential if the results are to inform patient safety, regulatory decisions, and sustainable material development.

## 7. Discussion and Future Perspectives

Despite increasing recognition of micro- and nanoplastics (MPs/NPs) in dentistry, the evidence base remains fragmented. Historically, dental research has emphasized bulk safety and mechanical performance of materials, while environmental sciences have rarely considered dentistry as a plastic pollution source. This disconnect has delayed targeted funding and interdisciplinary collaboration, leaving relevant findings scattered across fields [25].

Variability in dental materials and clinical practice further complicates the evidence. Polymer formulation, curing method, operator technique, and patient-specific factors such as chewing patterns and salivary pH all influence particle release [4,8,75,76]. For example, studies of orthodontic aligners have demonstrated brand-dependent differences in MP release under identical conditions [20], underscoring the need for systematic, comparative research.

Exposure patterns also present unique challenges. Dental MPs/NPs are released episodically, peaking during procedures such as polishing, debonding, or denture adjustment, rather than continuously [8,24]. Capturing these transient exposures requires repeated or continuous monitoring, which is technologically demanding. Translational gaps remain wide: most data come from in vitro or animal models [59,60,75], where particle characteristics and dosing differ substantially from clinical conditions. Ethical and practical constraints further limit direct human studies, since intentional patient exposure is not feasible [24,63,64].

Future progress depends on bridging these gaps. Standardized protocols for MP detection and quantification are urgently needed [72,73,74,75], alongside collaborative, multi-center studies that can improve comparability. Clinically, several mitigation strategies are already available, including high-volume suction, water cooling, effective ventilation, and proper waste management [76,77,78,79,80]. From a materials perspective, manufacturers should evaluate particle release with the same rigor applied to bulk strength or monomer leaching, and prioritize innovations such as wear-resistant polymers, biodegradable components, or protective coatings [77]. Finally, professional organizations are beginning to emphasize sustainability as a core ethical responsibility, aligning patient safety with environmental stewardship [81]. Translating these findings into practice requires concrete steps. Professional associations could incorporate MP/NP monitoring into clinical guidelines, while regulatory bodies such as ISO and ADA (American Dental Association) should consider integrating particle release testing into material safety standards. At the research level, priorities include developing standardized analytical methods, conducting longitudinal in vivo studies on patients with long-term restorations or prosthetics, and fostering collaborations between dental sciences, toxicology, and environmental health. In parallel, manufacturers should invest in designing biodegradable or low-shedding polymers that minimize both occupational and environmental burdens. These measures would ensure that evidence moves beyond hazard identification toward actionable risk assessment and sustainable solutions.

Overall, addressing MPs/NPs in dentistry requires both scientific rigor and professional accountability. By combining methodological innovation, clinical mitigation, and sustainable material development, the dental community can reduce risks for patients, practitioners, and the environment alike.

## 8. Conclusions

Dental procedures and materials are a source of micro- and nanoplastic release, yet the magnitude of exposure and its health implications remain uncertain. Current evidence is limited by analytical challenges, variability in materials and techniques, and gaps between laboratory findings and real-world conditions.

Advancing this field requires standardized methodologies, interdisciplinary research, and innovation in material science. At the same time, clinicians can already adopt simple mitigation strategies to reduce exposure during routine practice.

By combining rigorous science, sustainable material development, and responsible clinical practice, dentistry can address micro- and nanoplastics proactively—protecting both patient health and the environment.

## Data Availability

No new data were created or analyzed in this study. Data sharing is not applicable to this article.
